# Training, care delivery, and research in physiotherapy in sub-Saharan French-speaking Africa

**DOI:** 10.4102/sajp.v79i1.1932

**Published:** 2023-09-19

**Authors:** Oyéné Kossi

**Affiliations:** 1 ENATSE, National School of Public Health and Epidemiology, University of Parakou, Parakou, Benin; 2 Department of Neurology and NeuroRehabilitation, University Hospital of Parakou, Parakou, Benin

## Context

Over recent decades, the burden of diseases has shifted from communicable to non-communicable diseases with low- and middle-income countries (LMICs) bearing a huge part of this burden. The shift in the burden of diseases, especially the share in LMICs is thought to be driven by rapid population ageing, accompanied by a rise in physical and mental health challenges, injuries, in particular road traffic accidents, and comorbidities that occur in these countries (Yusuf et al., 2001).

Sub-Saharan French-speaking Africa (SSFA) is a group of 19 LMICs with a population over 345 million people (Table 1). In SSFA, the first training and physiotherapy care initiatives date from the 1970s with the opening of training schools for massage-physiotherapists in Lomé and in Kinshasa. In 1992, the Senegalese state undertook major reforms in the field of paramedical training with the creation of the National School of Health and Social Development establishing various departments including a physiotherapy department. However, the most impactful programme in the region would be the one that started in Benin in 1991. In this programme, the Belgian Association for the Promotion of Education and Training Abroad, the Wallonia Brussels International Federation and the Catholic University of Louvain have partnered to support the implementation of three development programmes of Physiotherapy and Physical Rehabilitation Medicine (PPRM) in Benin (1991), in Burundi (2008), and in Burkina Faso (2014). This highly successful programme enabled the creation of three reference centres in PPRM as well as three training schools in the capitals of the aforementioned countries. Several physiotherapy services were subsequently established within these countries.

**TABLE 1:** Density of training programmes and practising physiotherapists in sub-Saharan French-speaking Africa.

Country	Population in 2021 (million)	Number of training programmes	Number of training programmes per 100 000 population	Levels of training currently available	Practising physiotherapists in the country	Practising physiotherapists per 100 000 population
Benin	13	1	0.008	Bachelor, Certificate, Master	250	1.15
Burkina Faso	22.1	1	0.005	Bachelor	30	0.14
Burundi	12.55	1	0.008	Bachelor	150	1.20
Cameroon	27.2	6	0.02	Bachelor, Master	250	0.92
Central African Republic	4.5	0	0	NA	13	0.29
Comoros	0.81	0	0	NA	NA	NA
Congo	5.84	0	0	NA	15	0.26
Democratic Republic of the Congo	95.89	6	0.006	Bachelor, Master	1300	1.36
Djibouti	1.11	0	0	NA	NA	NA
Ivory Coast	27.48	1	0.004	Bachelor, Master	190	0.69
Gabon	2.34	3	0.128	Bachelor	50	2.14
Guinea	13.53	0	0	NA	10	0.07
Madagascar	28.92	1	0.003	Bachelor	200	0.69
Mali	21.9	1	0.005	Bachelor	180	0.65
Niger	25.25	1	0.004	Bachelor, Master	80	0.32
Senegal	16.88	1	0.006	Bachelor, Master	120	0.71
Seychelles	0.1	0	0	NA	NA	NA
Tchad	17.18	0	0	NA	20	0.17
Togo	8.65	1	0.012	Bachelor	400	4.62

**TOTAL**	**345.23**	**24**	**0.007**	**NA**	**3487**	**1.01**

NA, not available.

Despite all these efforts, the provision of physiotherapy services in SSFA is far from meeting the needs of populations not only from a quantitative point of view but also from a qualitative one. Recently, the World Health Organization (WHO) expressed deep concern that rehabilitation needs are largely unmet globally and that in many countries more than 50% of people do not receive the rehabilitation services they require. In some African countries, between 62.5% and 82.5% of people who need physiotherapy services do not benefit from them (Kamenov et al., 2019). Within the ministries of health, the priority given to physiotherapy and its planning are limited. Currently, the SSFA region has the lowest density of physiotherapy workforce compared with other regions in the world. In this scientific letter, I seek to analyse the current challenges in training, care delivery, and research in physiotherapy in SSFA and to recommend urgent actions.

## Training of physiotherapists in sub-Saharan French-speaking Africa

The first challenge to fill the gap of the low rate of physiotherapy workforce per population in the Africa region is to establish training programmes. Table 1 gives the density of training programmes and practising physiotherapists per population in SSFA. Information contained in Table 1 was obtained from the primary contacts of the World Physiotherapy member organisations by means of an electronic data capture form. So far, we have in total roughly 24 entry level education programmes in physiotherapy in SSFA for a population of more than 345m people. While the entry level is a bachelor level in all countries, several master’s programmes in physiotherapy are available. Unfortunately, so far, 6 (31.58%) out of the 19 countries of the region do not have any training programmes in physiotherapy. In addition, only Cameroon, Democratic Republic of the Congo, and Gabon have more than one entry level training programme (six in Cameroon and Democratic Republic of the Congo, and two in Gabon). Therefore, in the SSFA we have on average 0.007 training programmes per 100 000 population, which is very low compared with other regions or countries. For example, in Afghanistan there are 13 entry level education programmes in physiotherapy for 40.1m population (ratio: 0.032/100 000), in Belgium 17 for 11.59m (ratio: 0.147/100 000), in Australia 50 for 25.69m population (ratio: 0.32/100 000) (World Physiotherapy, 2022).

## Physiotherapy care delivery in sub-Saharan French-speaking Africa

Physiotherapy can be provided in many different settings, from hospital settings to private clinics or in community settings, such as in an individual’s home (Noukpo et al., 2022). As such, physiotherapy is recognised by WHO as an essential health strategy for achieving universal health coverage, increasing health and well-being, improving quality of life, delaying the need for long-term care and empowering people with disabilities to achieve their full potential and to participate in society.

As reported by World Physiotherapy, the Africa region continues to have the lowest ratio of physiotherapy workforce per population (World Physiotherapy, 2022). In 2022, the average number of physiotherapists per 100 000 population was 2.3 in Africa compared with 14.7 in the Asia Western Pacific region and 132 in Europe. In SSFA we found a ratio of 1.01 physiotherapist per 100 000 population (Table 1), the only one exception being Togo where we found a ratio of 4.62 physiotherapists per 100 000 persons.

Moreover, within each country there is a huge disparity of the physiotherapy workforce among regions. In most cases, physiotherapists are concentrated in the capitals and large cities, small towns and remote areas being left behind in terms of physiotherapists’ services. To cope with this situation, two solutions seem possible. Firstly, health and social authorities should provide support for the installation of physiotherapists in disadvantaged areas, which could help to resolve the differences between urban and rural or remote areas. This support could be in terms of: (1) material support for installation, (2) reduction or cancellation of taxes, (3) financial premium for installation, among others. Secondly, telerehabilitation and the use of affordable mobile health technologies are promising opportunities to overcome the scarcity of physiotherapists in remote or rural areas (Bonnechère et al., 2022a, 2022b; Brigo et al., 2022; Rintala et al., 2022). Especially, smartphones are expanding everywhere including in low resource areas. Therefore, smartphone-based applications might be good opportunities to supply or complement rehabilitation in remote or disadvantaged areas. A project is being developed in Lubumbashi (Democratic Republic of the Congo) on the use of virtual reality in the rehabilitation of children with sickle cell disease who are victims of stroke (Boma et al., 2023). For children and young people this type of technology is appropriate because it combines playful aspects as well as exercises aimed at improving both physical and cognitive functions. The only limitation is that such technology is not affordable for those with low or middle incomes; therefore, it is difficult to consider large-scale use.

In 2015, the United Nations adopted 17 Sustainable Development Goals (SDGs), as a universal call to action to end poverty, protect the planet, and ensure that by 2030 all people enjoy peace and prosperity. Goal 3 aims to ensure healthy lives and promote well-being for all, at all ages, which is essential to sustainable development. Incorporating physiotherapy services into the package of essential services, along with disease prevention, health promotion, treatment and palliative care would be an essential part of achieving target 3 of the SDGs.

## Research in physiotherapy in sub-Saharan French-speaking Africa

**FIGURE 1:**
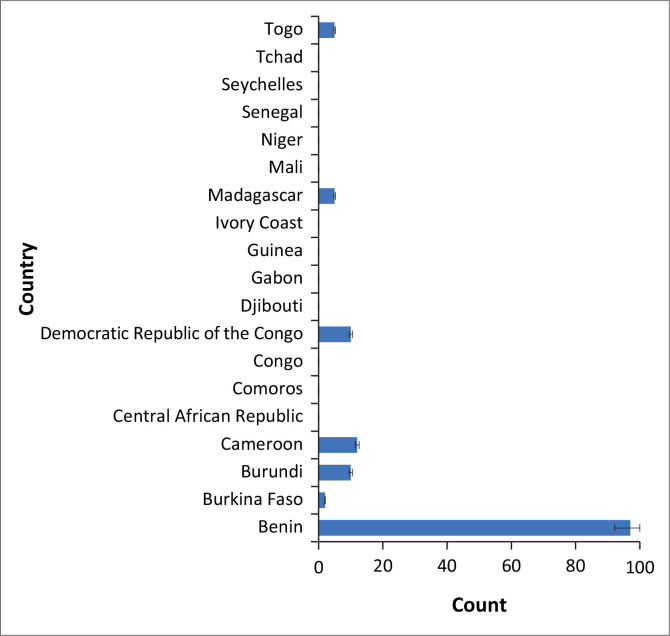
Number of scientific publications on physiotherapy in sub-Saharan French-speaking Africa.

Despite its utmost importance, evidence-based practice (EBP) is not well developed and implemented in SSFA. I conducted a literature search in PubMed/Medline, Web of Science, Science Direct, Google Scholar, and ResearchGate databases from inception to 15 June 2023. The search strategy combined keywords ‘physiotherapy’, ‘rehabilitation’, ‘physical therapy’, ‘Africa’. Articles were included if they fulfilled the following criteria: (1) at least one of the authors was a physiotherapist; (2) at least one of the authors’ affiliations was located in SSFA; (3) in the case of multiple authors of a publication including physiotherapists with affiliations located in different SSFA countries, the publication was attributed to the author with the highest rank in authorship. To date, in SSFA around 150 scientific publications on physiotherapy have been published and are available in electronic databases, most of which are observational studies. I found two randomised controlled trials in Burundi and in Benin (Niama Natta et al., 2021; Nindorera et al., 2023). I also found one study on a participation outcome measure developed and validated in Benin and Burundi (Kossi et al., 2018). Figure 1 gives the total number of articles identified per country. Most (63.31%) of the identified publications involved physiotherapists affiliated to a Beninese institution.

Since 2017 physiotherapists in the region are more and more actively involved in World Physiotherapy conferences. In 2022, for the first time the conference of the African region of World Physiotherapy was held in a French-speaking country (Benin). Thanks to North–South collaborations, physiotherapists from several countries in the region are undertaking PhD studies and are publishing their work. This trend needs to be reinforced not only through South–South collaborations but also through the effective integration of physiotherapist researchers into the body of researchers in their countries and their full integration in the academic body of the physiotherapy training programmes in the region. This will contribute to raising the level of training of physiotherapists and promote high-quality physiotherapy research in the region, including health policy and systems research.

## Call for action

Considering the aforementioned, I call for urgent action in the following five areas:

- Regarding the health and demographic trends in the region, there is an urgent need for the training of more practising physiotherapists.
- Physiotherapy must be brought closer to communities and patients’ homes by supporting the installation of physiotherapists in disadvantaged, rural or remote areas, and by promoting telerehabilitation and the use of mobile technologies in physiotherapy.
- Physiotherapy should be incorporated into universal health coverage so that no one needing rehabilitation is left behind.
- Solid networks and partnerships in physiotherapy should be established within the region including with non-French speaking countries.
- It is also of utmost importance to strengthen research capacity and expand the availability of robust EBP in physiotherapy in the region.

## Conclusion

Sub-Saharan French-speaking Africa needs to respond to the WHO rehabilitation 2030 call for action. Urgent and concerted actions are needed to expand the quantity and the quality of rehabilitation services in the region. There is also an urgent need to establish and/or strengthen networks and partnerships not only within the region but also with high-income countries to establish and expand strong scientific evidence adapted to local socioeconomic and cultural realities.
